# Stereotactic Body Radiation Therapy (SBRT) for Prostate Cancer in Men With a High Baseline International Prostate Symptom Score (IPSS ≥ 15)

**DOI:** 10.3389/fonc.2020.01060

**Published:** 2020-07-03

**Authors:** Nima Aghdam, Abigail Pepin, David Buchberger, Jason Hirshberg, Siyuan Lei, Marilyn Ayoob, Malika Danner, Thomas Yung, Deepak Kumar, Brian T. Collins, John Lynch, Shaan Kataria, Simeng Suy, Sean P. Collins

**Affiliations:** ^1^Department of Radiation Medicine, Georgetown University Hospital, Washington, DC, United States; ^2^George Washington University, School of Medicine and Health Sciences, Washington, DC, United States; ^3^University of Cincinnati College of Medicine, Cincinnati, OH, United States; ^4^Arizona College of Osteopathic Medicine, Glendale, AZ, United States; ^5^Julius L. Chambers Biomedical Biotechnology Research Institute, North Carolina Central University, Durham, NC, United States; ^6^Department of Urology, Georgetown University Hospital, Washington, DC, United States

**Keywords:** prostate cancer, SBRT, cyberknife, common toxicity criteria (CTC), quality of life, EPIC, IPSS

## Abstract

**Background:** Patients with a high pretreatment IPSS may have higher rates of late urinary morbidity after radiation therapy for prostate cancer (1). Stereotactic body radiation therapy (SBRT) delivers fewer high-dose fractions of radiation, which may be radiobiologically favorable to the conventional low-dose external beam fractions. The urinary toxicity associated with SBRT, however, remains unclear in patients with a high IPSS (1). We report our experience using SBRT for localized prostate cancer in patients with pretreatment IPSS ≥ 15.

**Methods:** Localized prostate cancer patients with a pre-treatment IPSS ≥ 15 treated with SBRT at Georgetown University Hospital from 2009 to 2016 were included in this retrospective review of prospectively collected data. These patients were treated to 35–36.25 Gy in five fractions delivered via CyberKnife (Accuray Inc., Sunnyvale, CA). Urinary toxicity was assessed using the Common Terminology Criteria for Adverse Events version 4.0 (CTCAE v4). Urinary quality of life was assessed using validated questionnaires (IPSS and EPIC-26).

**Results:** 53 patients at a median age of 71 years (range 57–89 years) received SBRT with a minimum follow up of 3 years. The median prostate size was 37 cm^3^ (range 12–100 cm^3^) and 30.2% patients received ADT. The 3-years incidence rate of Grade 3 urinary toxicity was 7.5% with median time to toxicity of 2.9 years. There were no Grade 4 or 5 toxicities. A mean baseline IPSS score of 19.8 significantly decreased to 12.9 at 3 months post-SBRT (*p* = 0.002) and remained stable at 36 months (13.7). A mean baseline EPIC-26 obstructive/irritative score of 64.1 significantly improved to 80.2 at 3 months (*p* = 0.002). This improvement was maintained to 36 months. There was no significant change from the mean baseline EPIC-26 urinary incontinence score at any point during follow up.

**Conclusions:** SBRT for clinically localized prostate cancer was well-tolerated in men with baseline IPSS ≥ 15 (1). Grade 3 toxicities occurred but resolved with time. Our data suggest that poor baseline urinary function does not worsen following SBRT and may even improve. High baseline IPSS score should not be considered a contraindication to SBRT.

## Introduction

The typical EBRT treatment for localized prostate cancer involves fractionated radiation therapy using 1.8–2.0 Gy daily doses for 8 to 9 weeks. In general, the treatment is well-tolerated in men with both good and bad baseline urinary function (2, 3). However, some studies have shown that patients with poor baseline urinary function may experience higher rates of late GU toxicity (4–6).

Unfortunately, several weeks of daily treatment is burdensome to many men. Brachytherapy is an alternative therapeutic option for patients who desire a shortened course of treatment. However, brachytherapy may not be appropriate for all patients with an increased risk of urinary morbidity in men with a prior TURP, large prostate size (>50 cc), or high baseline lower urinary tract symptoms (IPSS ≥ 15) (7–15). The relationship between late urinary morbidity and high baseline IPSS has been extensively reported for brachytherapy with some studies reporting acceptable late toxicity in patients with high pretreatment IPSS (9, 16). Pharmacologic interventions such as neoadjuvant androgen deprivation therapy (ADT) could reduce the prostate volume and improve urinary symptoms prior to brachytherapy; however, this comes at the expense of symptomatic hypogonadism that may take an extended period of time to resolve (17, 18). Alternatively, prophylactic alpha antagonist usage may prevent urinary morbidity (19).

The radiobiological favorability of larger fraction sizes coupled with the economic and convenience benefits of reduced treatment regimens make shorter EBRT fractionation approaches appealing (20–23). Over the past decade, moderately-hypo-fractionated (2.4–3.4 Gy per fraction) and ultra-hypo-fractionated (stereotactic body radiation therapy or SBRT) forms of radiation therapy have become accepted treatment approaches and are increasingly in use (24). Some, but not all, randomized studies have shown increased GU morbidity in men treated with moderately hypofractionated radiation therapy (25–29). Specifically, patients with poor baseline urinary function were vulnerable and had increased incidence of ≥2 GU toxicity in one study (30).

Multiple ultra-hypo-fractionated studies have demonstrated equivalent to superior rates of biochemical control in comparison to other definitive options, with comparable toxicity profiles (31–36). Recently, results from the two large-scale randomized trials directly comparing SBRT to conventionally fractionated radiation therapy have also been published (37, 38). The HYPO-RT-PC trial showed no significant difference in 5-years biochemical or clinical failure between the two modalities (84% in both arms). While there was a small increased rate of transient acute urinary toxicity reported in the SBRT group, there was no difference in long-term toxicity profiles (37). However, the PACE-B trial suggested no difference between SBRT and conventionally fractionated/hypo-fractionated approaches in terms of acute toxicity (38).

Still, the question remains whether men with high baseline IPSS scores can safely be treated with SBRT (39). Despite our understanding of the role of high IPSS in context of patient selection for brachytherapy, quality of life in patients treated with SBRT with poor baseline urinary function (IPSS ≥15) remains unclear. The purpose of this study was to evaluate the influence of a poor baseline urinary function on urinary morbidity in patients treated with SBRT.

## Methods

This study is a retrospective review of prospectively collected data from 53 consecutively treated patients with a pre-SBRT IPSS ≥ 15, who received SBRT at Georgetown University Hospital for localized prostate cancer, from January 2009 to December 2016. This IPSS cutoff of 15 was utilized due to its known association with increased urinary toxicity following brachytherapy (16). Risk stratification was defined using the D'Amico classification (40). Clinical stage was assigned according to the 6th edition of the American Joint Committee on Cancer definitions (41). Exclusion criteria included <3 years of clinical follow-up or prior pelvic irradiation. An institutional review board approval was obtained for this analysis (IRB#: 2009-510).

### Treatment Planning and Delivery

The fiducial placement and CT/MRI simulation procedures has been previously described (42). Treatment planning and dose constraints have been previously reported (31). Patients were treated to a dose of 35–36.25 Gy in five fractions via CyberKnife (Accuray Inc., Sunnyvale, CA).

### Pretreatment Assessment and Follow-Up

The International Prostate Symptom Score (IPSS) and the Expanded Prostate Cancer Index Composite (EPIC-26) short form were used to evaluate urinary symptomatology prior to treatment and during subsequent follow-up visits (43, 44). Initial assessments were performed on the day of initial consultation. Follow-up questionnaires were completed at start of treatment, 3-months post-treatment, every 3-months to 1-year post-treatment, and every 6 months thereafter. IPSS alternatively is scored between 0 and 35 with higher scores suggesting worse symptoms. The EPIC urinary domain is scored on a range from 0 to 100 with higher values representing more favorable urinary symptoms. Toxicities at each time point were scored using the CTCAE v4. The utilization of alpha_1_ antagonists was prospectively documented at each visit.

### Statistical Analysis

A QOL change of a ½ standard deviation (*SD*) from the baseline QOL score, defined as the minimal important difference (MID), was used to denote a clinically significant change in the QOL score (45). The two-sided paired Wilcoxon rank-sum test was used to calculate the significance of differences in the mean scores on follow-up as compared to the baseline values. Parameters were identified as significant if the two-tailed *p*-value was < 0.05. GraphPad Prism version 8.0.0 for Windows was used for analysis (GraphPad Software, San Diego, California USA).

## Results

With minimum follow up of 3 years and median follow up of 60 months (95% CI, 50.4–69.5), 53 men with pre-radiation IPSS ≥ 15 were treated with SBRT at our institution between January 2009 and December 2016 met the inclusion criteria for this analysis. Patient characteristics are represented in Table 1. The median age was 71 years (range: 57–89) and 18.9% were obese (BMI > 30). The cohort was diverse with 50.9% of the population identifying as white and 39.6% as black. Comorbid conditions were common with 43.4% of the patients having a CCI of ≥ 2 and 41.5% were on anticoagulation. Approximately 43% of patients had palpable disease (T2–T3). The median prostate volume was 37.3 cm^3^ (range: 12–100 cm^3^) with 13.2% of the patients having had a prior TURP. There were 31 intermediate-risk and 7-high risk patients in the cohort. Alpha-1-antagonists were utilized at the time of initial consultation in 39.6% of patients. Approximately 30% of the cohort received neoadjuvant androgen deprivation therapy for 3 months prior to SBRT. A roughly equal amount of the cohort were treated with 36.25 and 35 Gy in five fractions (54.7% vs. 45.3%, respectively).

**Table 1:** Patient, tumor, and treatment characteristics.

	***n* = 53**
**Age (years)**	
Median (range)	71 (57–89)
**Race**	
White	27 (50.9%)
Black	21 (39.6%)
Other	5 (9.4%)
**Gleason score**	
6	19 (35.8%)
7	30 (56.6%)
8–9	4 (7.5%)
**T-stage**	
T1c	30 (56.6%)
T2	22 (41.5%)
T3	1 (1.9%)
**CCI**	
0	11 (20.8%)
1	19 (35.8%)
≥2	23 (43.4%)
**Risk group**	
Low	15 (28.3%)
Intermediate	31 (58.5%)
High	7 (13.2%)
**Hormone treatment**	
Yes	16 (30.2%)
No	37 (69.8%)
**Anticoagulation/Antiplatelet**
Yes	22 (41.5%)
No	31 (58.5%)
**Dose (Gy)**	
35	24 (45.3%)
36.25	29 (54.7%)
**Pretreatment** **α-antagonist**	
Yes	21 (39.6%)
No	32 (60.4%)
**BMI**	
18.5–24.9	17 (32.1%)
25–29.9	26 (49.1%)
> 30	10 (18.9%)
**Pretreatment TURP**	
Yes	7 (13.2%)
No	46 (86.8%)
**Prostate volume (cc)**	
Median (range)	37.3 (12–100)

In the 3 years following treatment, the actuarial incidence rate of Grade 3 toxicities was 7.5% (*n* = 4) (Table 2). This included two patients who required TURP for urinary retention, one patient who required intermittent catheterization secondary to urinary retention, and one patient who required fulguration for hematuria. Median time to grade 3 toxicity was 35 months (CI 95% 21.0–52.4 months) with a range of 16–56 months. There were no Grade 4 or 5 toxicities.

**Table 2:** Grade 3 GU toxicities.

	**Age**	**Charlson comorbidity index**	**IPSS score at initial consult**	**Baseline prostatic volume (cc)**	**Coagulation use**	**History of prior TURP**	**Intervention performed**
1	72	0	24	78	Yes	No	Intermittent Catheterization
2	68	1	21	12	No	Yes	TURP
3	66	2	20	36	Yes	No	TURP
4	65	2	21	43	No	Yes	Fulguration

**Figure 1:**
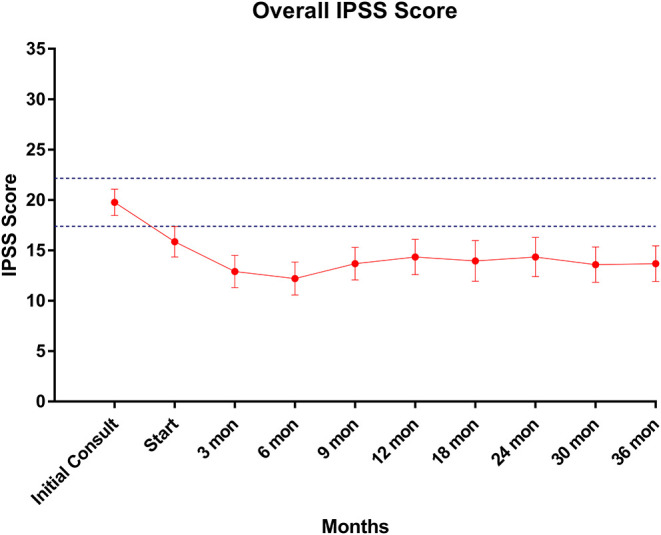
IPSS score trend post-SBRT. Error bars represent 95% CI. Dashed lines represent 0.5 *SD* above and below baseline.

**Figure 2:**
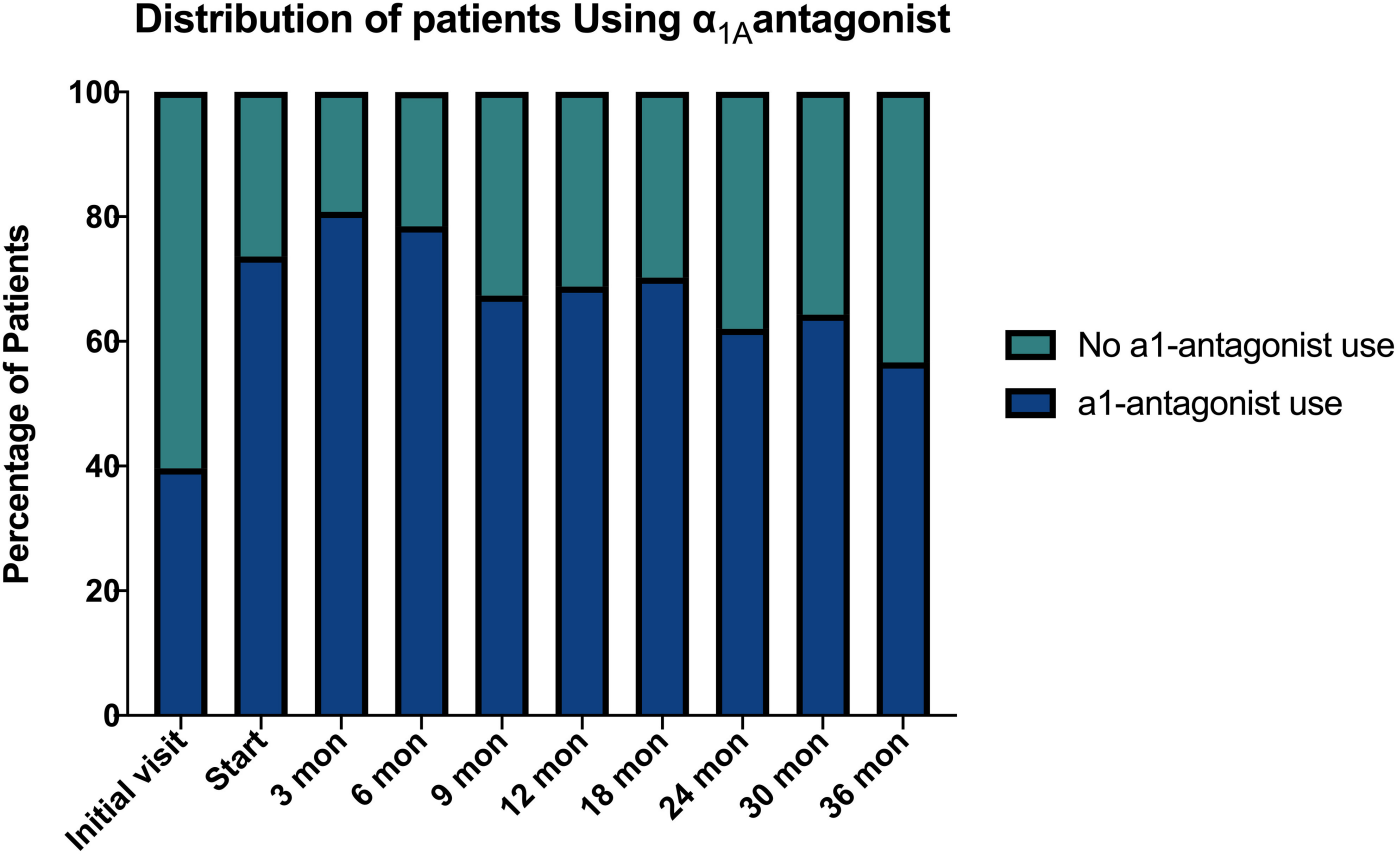
a1-antagonist use over time.

**Figure 3:**
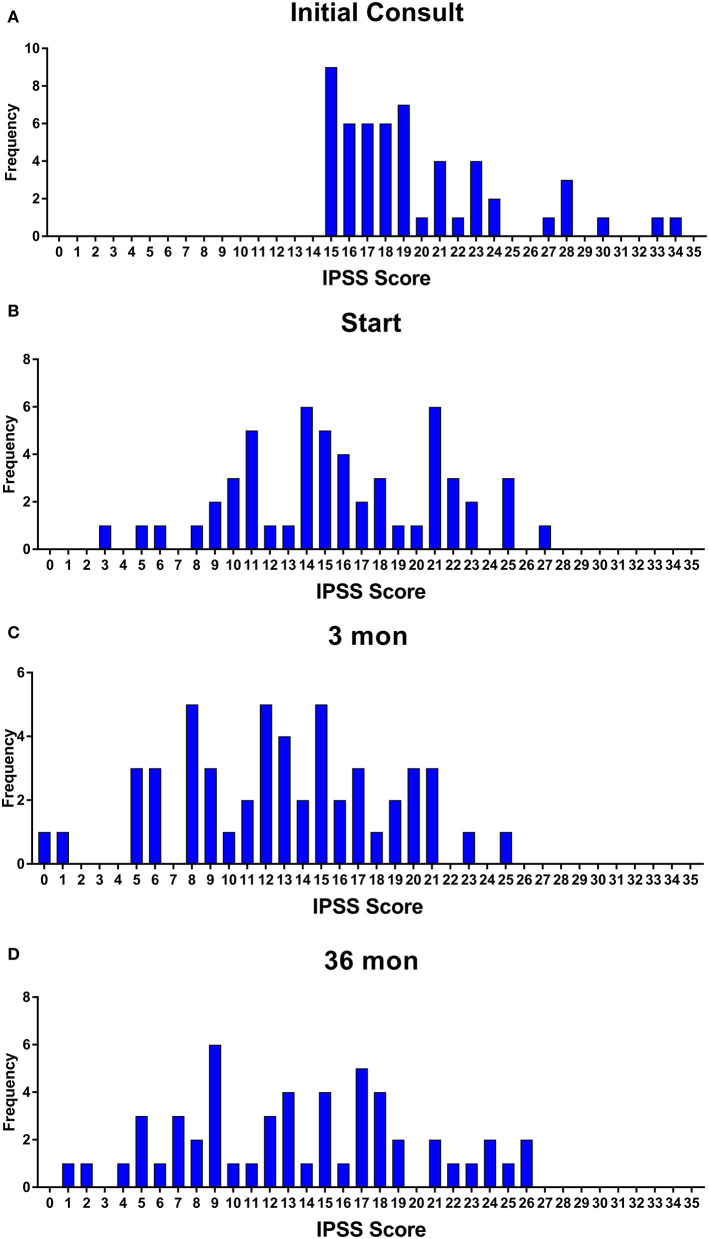
Incidence of IPSS score at **(A)** initial consult, **(B)** start of treatment, **(C)** 3 months, and **(D)** 36 months.

Baseline quality of life scores are shown in Table 3. The mean IPSS scores over 3 years are reported in Figure 1. At initial consult, the mean IPSS score was 19.8 (range of 15–34). At start of SBRT treatment, the mean IPSS score significantly decreased to 15.8 (range 3–27). This decline was both statistically (*p* = 0.002) and clinically significant (MID 2.4). This corresponded with increasing percentage of people using a1-antagonists from initial consult (39.6%) to start of treatment (73.6%) (Figure 2). The average IPSS scores once again significantly declined to 12.9 (range 0–25) at 3 months post-SBRT (*p* = 0.002, MID 2.4). This corresponded with 80.8% of the patients utilizing a1-antagonists 3 months post-SBRT. The mean IPSS remained stable at 13.7 in 36 months (range 1–26) (*p* = 0.002, MID 2.4). All individuals had moderate to severe urinary symptoms at baseline (Figure 3A). At the start of treatment, already three of those individuals improved to have only mild urinary symptoms (Figure 3B). At 3 months, eight patients only mild urinary symptoms (Figure 3C). By 36 months, 10 total individuals improved to only have mild urinary symptoms (Figure 3D), representing an improvement from baseline.

**Table 3:** Baseline toxicity scores by IPSS and EPIC-26 urinary incontinence and irritative/obstructive domains.

	**% Patients (n=53)**	**Mean**	***SD***	**MID**
**Baseline IPSS score**				
0–7 (mild)	0% (0)	20	4.8	2.4
8–19 (moderate)	64.2% (34)			
>20 (severe)	35.8% (19)			
**Baseline EPIC-26 summary score**				
Urinary incontinence domain		79.6	20.2	10.1
UUrinary irritative/obstructive domain		64.1	18.4	9.2

A multivariate logistic regression was conducted to determine other patient and treatment specific factors, which may contribute to improvement in IPSS score after SBRT. The considered variables included: race, age, BMI, SBRT dose level, prostate volume, and pretreatment α-antagonist use. None of these variables appear to significantly contribute to the improvement of IPSS score after SBRT (Supplementary Table 1).

**Figure 4:**
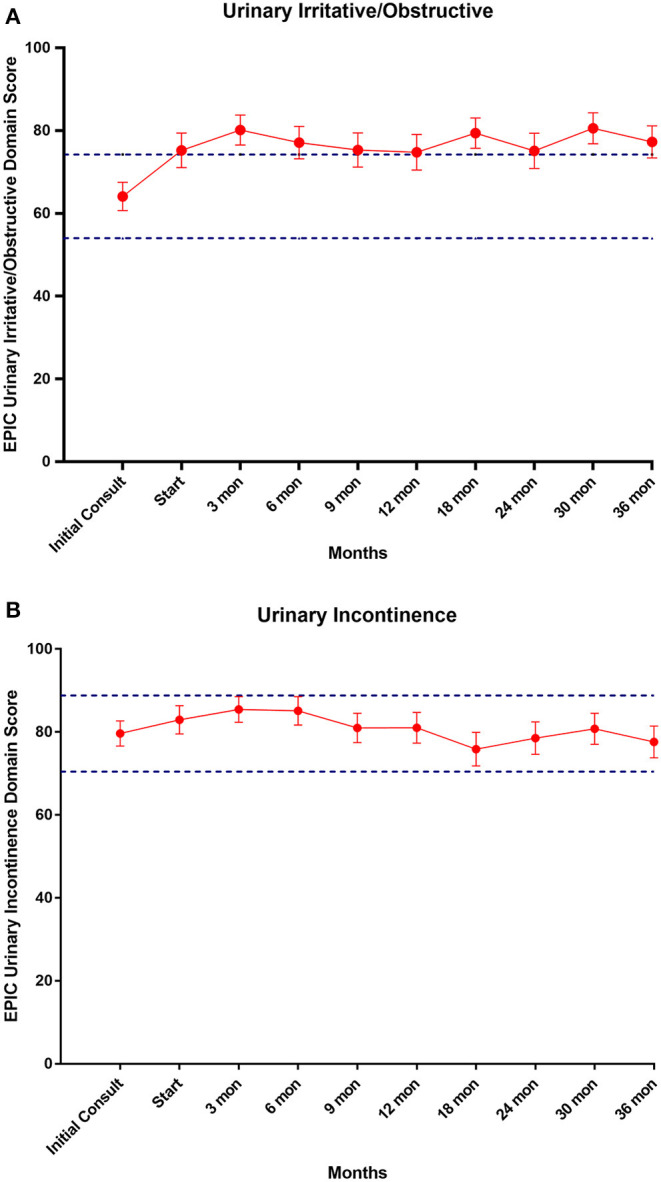
**(A)** Mean EPIC-26 urinary irritative/obstructive score trend post-SBRT, **(B)** mean EPIC-26 urinary incontinence score trend post-SBRT. Error bars represent 95% CI. Dashed lines represent 0.5 *SD* above and below baseline.

EPIC-26 scores were divided into irritative/obstructive and incontinence domains. Analyses of urinary function health related quality of life are shown in Figure 4. At the time of initial consultation, our patient population had low baseline irritative/obstructive and incontinence scores: 64.1 and 79.6, respectively (Table 3). The irritative/obstructive domain clinically significantly improved from initial consult to start of treatment (mean change from baseline, +11.1). The domain improved at 3 months (mean change from baseline, +16.0) (*p* = 0.002) and remained stable to 3 years (mean change from baseline, +13.1) (*p* = 0.002). These changes were both statistically and clinically significant (MID = 9.2). The incontinence domain also improved at 3 months (mean change from baseline, +5.8) (*p* = 0.0020) remained stable at 36 months (mean change from baseline,−2.0) (*p* = 0.0020). These changes while statistically significant did not meet the criteria for clinical significance (MID = 10.1).

## Discussion

The concern for treating patients with high IPSS scores stems from the experiences observed in patients treated with brachytherapy (7). The ABS guidelines currently advise caution for treatment of patients with high IPSS using brachytherapy based on previous studies documenting increased frequency of acute urinary obstructive and irritative symptoms requiring catheterization or other interventions in this population (7, 46). The relationship between high baseline IPSS and conventionally fractionated external beam radiation related toxicity has not been as widely documented. A study reviewing the genitourinary toxicity of patients treated with EBRT stratified by IPSS score >15 demonstrated that patients with higher baseline IPSS had increased rate of GU toxicity (Table 4) (2). Despite this, their findings suggested that men with higher baseline IPSS can have some improvement in urinary function following treatment.

**Table 4:** Summary of grade 3 toxicities reported for various radiation techniques in individuals with high baseline IPSS scores.

**Author**	**Institution/Trial**	**Technique**	**Dose (Gy)**	**Median follow-up (years)**	**No. of high IPSS patients**	**Gr 3 genitourinary (%)**
Malik et al. (1)	University of Chicago	EBRT	68.5–76.4 Gy	3.3	80	6.3%
Our unselected population (46)	Georgetown University	SBRT	35–36.25 Gy	2.3	100	1%
Our Population	Georgetown University	SBRT	35–36.25 Gy	5	**53**	7.5%

Our findings are consistent with previously published findings in conventional radiation, and the trends mimic those of other prostate cancer SBRT toxicity/QOL data. SBRT at our institution for the treatment of prostate cancer in patients with poor baseline urinary function was well-tolerated. However, the incidence of Grade 3 toxicity was higher than previously reported in unselected populations undergoing prostate SBRT (31). The etiology of this increased toxicity was likely multifactorial in origin including that which we selected for: poor baseline urinary status. Additional factors such as increased age at treatment, high comorbidity scores and high anticoagulant usage in our patient population likely contributed to increased risk of radiation therapy related toxicity (Table 1). Despite this, our toxicity levels are comparable to other radiation modalities in high baseline IPSS patients (see Table 3). Malik et al. found the rate of late grade 3 urinary toxicities occurred in 6.3% of patients with high baseline IPSS treated with conventionally fractionated EBRT (2). We found the rate of late grade 3 urinary toxicities to be 7.5% in our patient population. Significant toxicity was found to be acceptable in our patient population.

In 2013, King et al. published early post-SBRT QOL results from a multi-institutional consortium of prospective trials (47). Using the EPIC questionnaire, they analyzed 864 localized prostate cancer patients treated with SBRT across four institutions with a median follow-up of 36 months. Similar to the results in our unselected patients, they found a decline in urinary function over the first 3 months following treatment (quantified by a decrease in EPIC scores), which was followed by a recovery to pre-SBRT levels by 6-months, and an improvement in urinary symptoms from baseline by 3-years post-treatment (47, 48). After stratifying by urinary function, they also observed that symptomatology in patients with poorer urinary performance at baseline (in the lower 25th percentile) followed this trend, with recovery to “better than baseline” by 3 years post treatment.

Surprisingly, in this study we saw a significant improvement in IPSS score in the 3 months following SBRT which was maintained up to 3 years post treatment. This improvement in urinary function is likely multifactorial in origin. Treatment with neoadjuvant ADT and the initiation of an alpha-blockers likely contribute to improvement from initial consult to 1st day of treatment. Improvement in urination from the 1st day of treatment to 3 months post-SBRT is more difficult to explain. One hypothesis is that decreased cancer burden secondary to radiation therapy may improve urinary symptoms (49). The improving IPSS scores did mirror early PSA declines lending credence to this hypothesis. Alternatively, decreased inflammatory cells in the prostate in the months following radiation treatment may result in improvement in overall symptomatology (50). Tan et al. previously documented the relationship between moderate to severe urinary symptoms and higher scores on the Intolerance of Uncertainty (IUS) scores (51). As such, reduced prostate cancer specific anxiety may have decreased urinary symptoms in this population.

To more comprehensively evaluate urinary function in patients with high baseline IPSS, we evaluated changes in the EPIC score following SBRT. As expected, the baseline irritative/obstructive and incontinence EPIC scores in this group were lower than the baselines in our unselected cohort of SBRT patients (31). The obstructive/irritative score clinically significantly improved 3 months post SBRT (MID = 9.2) and this increase was maintained for the duration of the study. The 11–16 point increase represents a large improvement in obstructive/irritative symptoms (52). In this patient population with poor baseline urinary function, it is reassuring that there was no clinically significant increase in the urinary incontinence score at any point during follow up.

Limitations of this study include the small sample size, retrospective nature of the analysis and lack of control cohort. Additionally, it is possible to evaluate some of these symptoms using urodynamic studies. This would be the gold standard in determining an objective measure of both obstruction and incontinence. Future efforts must be directed to utilizing urodynamic testing in larger prospective studies.

## Conclusion

In patients with high baseline IPSS, prostate SBRT was well-tolerated. Grade 3 toxicities were modest. Overall urinary symptoms improve with significant improvement over time. Our institutional experience supports the effectiveness and safety of SBRT even for patients with poor baseline urinary function.

## Data Availability Statement

The datasets generated for this study are available on request to the corresponding author.

## Ethics Statement

The studies involving human participants were reviewed and approved by the Georgetown University Institutional Review Board. The patients/participants provided their written informed consent to participate in this study.

## Author Contributions

NA and AP were the lead authors, who participated in data collection, data analysis, manuscript drafting, table/figure creation, and manuscript revision. DB and JH participated in data collection and data analysis. TY and MA aided in clinical data collection. MD contributed to the study design and clinical data collection. SL developed the majority of patients' SBRT treatment plans and contributed to the data analysis and interpretation. SS is a senior author who organized the data and participated in its analysis. DK participated in data analysis and manuscript review. BC is a senior author who aided in drafting the manuscript. SC was the principal investigator who initially developed the concept of the study and the design, aided in data collection, and drafted and revised the manuscript. All authors contributed to manuscript revision, read, and approved the submitted version.

## Conflict of Interest

SC and BC serve as clinical consultants to Accuray Inc. The Department of Radiation Medicine at Georgetown University Hospital receives a grant from Accuray to support a research coordinator. The remaining authors declare that the research was conducted in the absence of any commercial or financial relationships that could be construed as a potential conflict of interest.

## Abbreviations

ADT: androgen deprivation therapy
CCI: Charlson Comorbidity Index
CT: computerized tomography
CTC: Common Toxicity Criteria
CTV: clinical target volume
DVH: dose-volume histogram
EBRT: external beam radiation therapy
EPIC: expanded prostate cancer index composite
GU: genitourinary
IMRT: intensity modulated radiation therapy
IPSS: international prostate symptom score
MID: minimal important difference
MRI: magnetic resonance imaging
QoL: quality of life
SBRT: stereotactic body radiation therapy.
