# Supplementary material for: Stereotactic Body Radiation Therapy (SBRT) for Prostate Cancer in Men With a High Baseline International Prostate Symptom Score (IPSS ≥ 15)
Source: Front Oncol. 2020 Jul 3;10:1060. doi: 10.3389/fonc.2020.01060 (PMC7350884; doi:10.3389/fonc.2020.01060)
Supplement: Supplementary file 1 [file Table_1.docx]

Supplementary Table 1. Multivariable analysis of factors effecting IPSS score improvement at 12 months after SBRT. The following variables were entered into the model: Race (White vs. Non-White), Volume (Above vs. less than or equal to 37.3 cc), Initial Flomax Use (Yes vs. No), Age (Above vs. less than or equal 71 years), Dose Level(35 vs. 36.25Gy), BMI (Above vs. less than or equal to 25).

| Multivariate Analysis of Factors Effecting IPSS improvement at 12 months | | | | |
| --- | --- | --- | --- | --- |
| Variables | Significance | Odds Ratio | 95% Confidence Interval | |
|  |  |  | Lower | Upper |
| Race | 0.988 | 0.99 | 0.278 | 3.524 |
| Prostate Volume | 0.273 | 1.946 | 0.592 | 6.395 |
| Initial Flomax use | 0.268 | 2.012 | 0.584 | 6.931 |
| Age | 0.92 | 1.066 | 0.307 | 3.704 |
| Dose Level | 0.771 | 1.212 | 0.332 | 4.43 |
| BMI | 0.394 | 1.811 | 0.463 | 7.089 |
